# The role of lipids in corneal diseases and dystrophies: a systematic review

**DOI:** 10.1186/s40169-017-0158-1

**Published:** 2017-09-01

**Authors:** Tyler G. Rowsey, Dimitrios Karamichos

**Affiliations:** 1 University of Oklahoma, College of Medicine, Norman, OK USA; 2 Department of Ophthalmology/Dean McGee Eye Institute, University of Oklahoma Health Sciences Center, Oklahoma City, OK USA

**Keywords:** Corneal lipidomics, Lipid-based therapy, Corneal diseases

## Abstract

Corneal diseases are an extensive cause of blindness worldwide and continue to persist as a challenging public health concern. Recently, various lipid-based therapies have been advocated for the modulation of corneal diseases; however, the number of studies is still very limited. Here we focus on developments and challenges on lipid-based therapies for dry eye disease, diabetic neuropathy, and Fuchs’ endothelial corneal dystrophy. All three diseases are highly prevalent conditions and involve corneal stress and inflammation. Lipid-based therapeutics discussed includes cyclooxygenase inhibitors, essential fatty acids, and resolvin analogs. Lipids also show increasing promise as biomarkers of disease and are explored in this review.

## Introduction

### Cornea structure

**Fig. 1:**
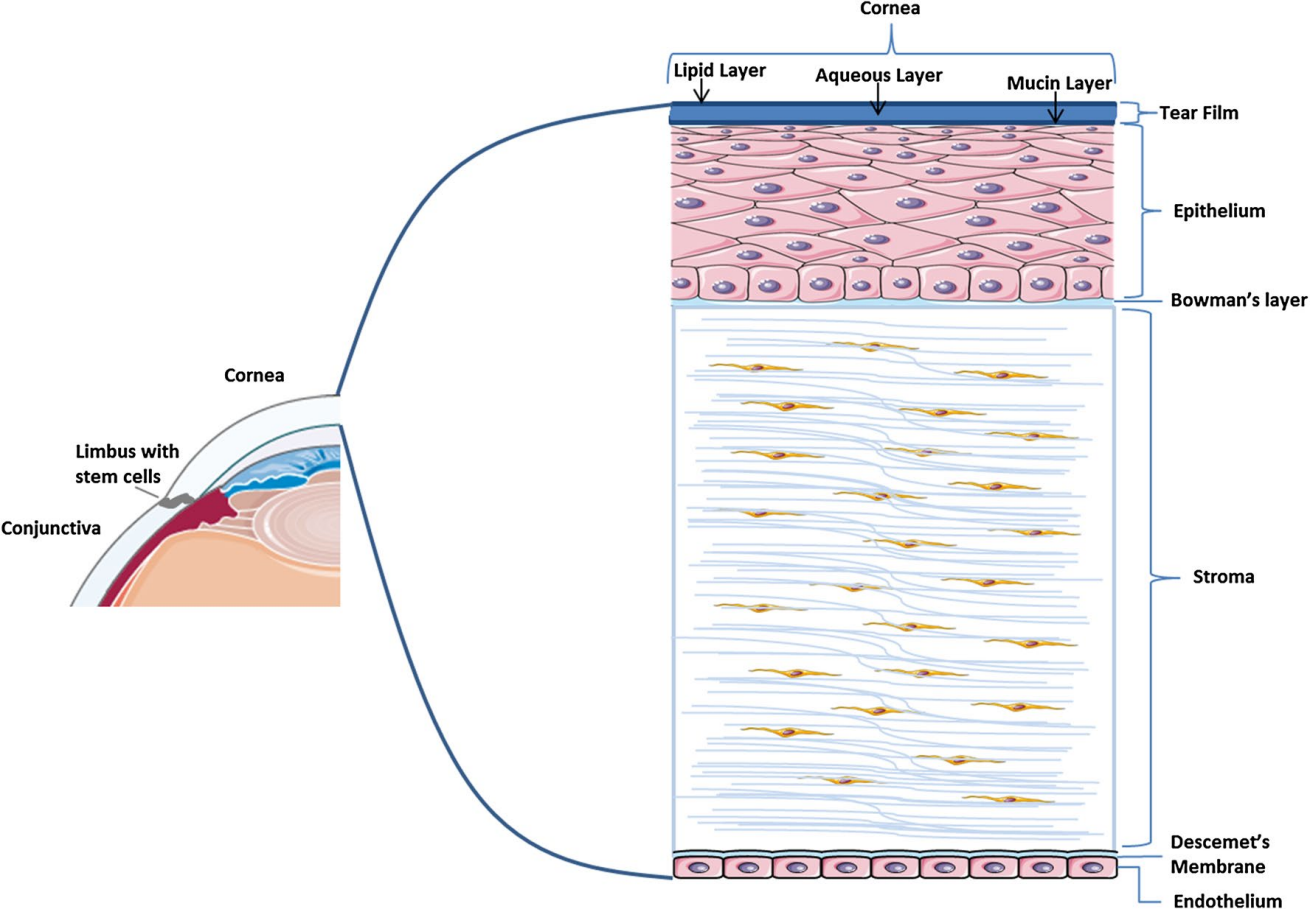
Structure of the cornea. Some images were provided by servier medical images

The cornea is composed of a clear, dome-shaped layer of tissue that provides transparency and light refraction to the eye [1–3]. The cornea, limbus, and bulbar and tarsal conjunctiva make up the ocular surface and function to protect the eye by providing the first line of defense against damage and infection [3, 4]. The cornea consists of five major layers including the epithelium, Bowman’s Layer, stroma, Descemet’s membrane, and endothelium (Fig. 1); it is approximately 500 μm thick and accounts for two-thirds of the refractive power of the eye [5]. The cornea is one of the most sensitive areas in the body, highly innervated, with a nerve density that tends to be around 300–600 times higher than that of the skin [3]. The peripheral cornea has sensory nerve fibers that have a myelinated shell. The central cornea, which is innervated by the ophthalmic nerve, tends to be less sensitive along the vertical meridian and more sensitive along the horizontal meridian [6]. The arrangement of the collagen layers in the stroma and the regular arrangement of collagen fibrils in the cornea are considered to be critical for the maintenance of corneal transparency [7].

### Damage to the cornea

When injury or disease proceeds, it can lead to corneal opacities or even blindness through the disordering of the extracellular matrix [7]. The corneal endothelial monolayer of cells is the primary contributor to the maintenance of corneal transparency. When the barrier functions of the endothelium are compromised, it results in a loss of visual acuity [8, 9]. Unfortunately, once there is damage to the cornea, it has proven to be complicated through countless studies to reverse this process.

Wound healing in the cornea is a diverse process involving many factors including cell death, migration, proliferation, differentiation, and extracellular matrix remodeling [10]. Similarities and differences are observed in the healing processes of corneal epithelium, stroma, and endothelium, as well as cell-specific alterations in each of the layers [10]. Damage to the cornea from diseases such as dry eye, corneal edema, diabetes mellitus, and dystrophies are prevalent conditions and often involve ocular surface stress and inflammation. One of the key features associated with these corneal diseases is oxidative stress [11]. Oxidative stress increases advanced glycation end product (AGE) accumulation and activation of Protein kinase C and the polyol pathway [12, 13]. Chronic inflammation of the ocular surface is common among many diseases such as dry eye disease [14–17]. The inflammatory process within the cornea can cause substantial irreversible damage to the corneal as well as the conjunctival epithelia, with subsequent visual loss [18]. The damage to the cornea from these diseases are an extensive cause of blindness worldwide and continue to persist as a challenging public health concern [19].

### Current treatments and lipids

The anatomical and physiological barriers that the cornea offers against the entrance of bacteria and other pathogens may also pose difficulties for the access and effectiveness of drugs. Current topical treatment options for ocular surface inflammation available today include mainly corticosteroid eye drops containing antibiotics, non-steroidal anti-inflammatory agents and cyclosporine A. Topical corticosteroids have shown through previous studies to have a rapid onset of action and efficacy when treating DED [20], but they have a limited range of effectiveness because of their potential side effects, which include posterior subscapsular cataract and increased intraocular pressure [21]. Other studies have found that these barriers may be overcome through the use of a variety of lipid based therapies.

Lipids are classified from a group of hydrophobic or amphiphilic small molecules composed of the carbanion-based condensation of thioester or isoprene groups; they include fatty acids, glycerololipids, sphingolipids, and sterols [22]. Lipids execute a variety of biological functions including cell signaling, energy storage, and maintenance of compartmental boundaries. In this review, we highlight the most recent developments in lipid-based therapies to modulate ocular surface inflammation as well as the potential for lipid-based molecules as a biomarker of disease in multiple ocular dysfunctions including DED, diabetic retinopathy, and Fuchs’ endothelial corneal dystrophy (FECD) (Table 1). Recent research has revealed the potential use of lipid therapeutics for the improvement of corneal damage due to dystrophies and disease.

**Table 1:** Lipid therapeutics/biomarkers available for corneal diseases

Disease	Lipid therapeutics/biomarkers
Dry eye disease [17]	Eicosapentaenoic acid, docosahexaenoic acid, and alpha linolenic acid, and resolvins have shown promise in improving symptoms of DED, matrix metalloproteinase-9 as an ideal biomarker due to its involvement in ocular surface inflammation
Diabetic neuropathy	Menhaden oil, daily injections of resolvin-D1, salsalate, enalapril, neuroprotectin D1, docosahexaenoic acid, as well as combination of enalapril, α-lipoic acid and menhaden oil have all shown to improve diabetic neuropathy
Fuchs’ endothelial corneal dystrophy	Mefenamic acid and nimesulide have shown to improve oxidative stress, ROCK inhibitor show potential for regenerative medicine, Diacylglycerophosphocholines, 1-ether, 2-acylglycerophosphocholines, eight sphingomyelins, and up to two long-chain highly unsaturated cholesteryl esters increased in the AH of FECD eyes, indicating potential oxidative stress markers

## Dry eye disease

Dry eye disease refers to a condition in which the individual does not produce enough quality tears to lubricate and nourish the eye [23]. Tears are needed to maintain the health of the front surface of the eye and also to allow clear vision. DED is a complicated disease of the ocular surface, which includes any of the following symptoms: visual disturbances, eye discomforts, and dryness due to tear film instability [24]. Pathogenesis for DED includes increased osmolarity of the tear film and inflammation of ocular surfaces and lacrimal glands [25]. It is clinically characterized into two separate subtypes: hyperevaporative DED with increased tear evaporation and aqueous-deficient DED with decreased tear secretion [26]. It can lead to visual loss, damage to the ocular surface, discomfort and overall reduction in the quality of life [25].

Due to the significant role meibomian glands play in providing lipids to the tear film, meibomian gland dysfunction (MGD) is one of the leading causes of DED [27]. A decrease in MGD leads to an increase in the evaporation of tears from the ocular surface [28]. Meibum is secreted by the Meibomian gland, and it is a predominant source of lipids that are important for the maintenance of tear film stability [29]. DED is a common and often chronic problem, particularly in older adults. There are numerous new therapeutic approaches that are under development, including anti-inflammatory agents, secretory stimulants, and tear film stabilizers; as a result, as improved endpoints are incorporated into clinical trials, it is likely that multiple therapeutic agents will emerge in the foreseeable future [4]. One recently approved lipid based molecule for the treatment of DED is lifitegrast; it exerts its mechanism of action by preventing LFA-1/ICAM-1 from interacting which prevents T cell activation and recruitment [30]. This drug was approved for the treatment of DED in 2016 due to its excellent therapeutic efficacy as ophthalmic drops and its rapid onset of action [30].

### Omega-6 linolenic acid/omega-3 fatty acids

Artificial tears provide temporary symptomatic relief and are the most common therapy for DED, but they do not address any of the underlying pathogenic mechanisms that lead to DED. Inflammation plays a contributing role in the pathogenesis of DED, indicating the vast importance of lipid-based therapies to reduce inflammation of the ocular surface.

There are three ω-3 fatty acids that cannot be synthesized in the body and must therefore be supplemented in the diet; these include eicosapentaenoic acid (EPA), docosahexaenoic acid (DHA), and alpha linolenic acid (ALA) [31]. Prostaglandin metabolism is modulated through EPA and DHA through an anti-inflammatory prostaglandin synthesis due to the competitive inhibition of the arachidonic acid pathway [32]. Supplementation of ω-3 prevents the creation of any new ω-6 prostaglandin precursors, which in turn inhibits apoptosis of the secretory epithelial cells in the lacrimal gland and helps clear meibomitis; this allows for a healthier lipid layer to protect the cornea and tear film [33]. ALA is a polyunsaturated fatty acid (PUFA) that has shown the potential to improve dry eye symptoms through a dietary supplement due to its anti-inflammatory effects [34–36]. An in vivo study showed that n-3 PUFA has several mechanisms that contribute to its anti-inflammatory effects through inhibition of nuclear factor kappa B (NF-κβ) activation in an animal inflammation model as well as through regulation of eicosanoid metabolism [37]. Recent studies have also shown that the ocular surface epithelia and tears of patients with DED, as well as in many animal models have an enhanced expression of pro-inflammatory mediators which include adhesion molecules, protein matrix metalloproteinases, cytokines, and chemokines [38, 39]. DED patients, as well as those who suffer from Sjogren’s syndrome have also been found to have an increased production and activation of interleukin-1β (IL-1β), interleukin-6 (IL-6), interleukin-8 (IL-8), tumor-necrosis factor-α (TNF-α), and transforming-growth factor-β (TGF-β), indicating the significant therapeutic implications of treatment with ALA [40]. Interleukin-1α (IL-1α) is able to up-regulate TNF-α release as well as its own autocrine production [41]. TNF-α has been described as a key mediator in the pathogenesis of DED [42]. ALA treatment was shown to be an effective treatment for decreasing the corneal and conjunctival expression of IL-1α and TNF-α [43]. Rashid et al. also revealed that the treatment of ALA caused a nearly 100-fold increase in expression of interleukin-10 (IL-10) in dry eye conjunctiva [43]. IL-10 is produced by activated machophages and lymphocytes, and it acts to inhibit IL-1 and TNF production, which further contributes to the down-regulation of inflammation [43]. The PUFA pathways for n-3 and n-6 ω-fatty acids competitively utilize the same enzymes [44]. Topical ALA treatment led to significant decrease in dry eye signs and inflammatory changes at both cellular and molecular levels [43]. Findings demonstrated that ω-3 and ω-6 can act directly on immortalized human Meibomian gland epithelial cells to have positive impacts on the quality and quantity of intracellular lipids [31].

### In vivo studies

In order to demonstrate that an oral supplementation of ω-3 fatty acids, antioxidants, and vitamins improves dry eye symptoms, a large study was conducted to determine the effects of this treatment. One study population, which consisted of 1419 patients, included 74% women with a median age of 58.9 years showed dietary supplementation with ω-3 essential fatty acids, antioxidants, vitamins, and minerals were useful to improve dry eye symptoms [45]. To relieve ocular surface dysfunction associated with DED, these patients used artificial tears as well as attended routine daily practice [45]. Other positive effects shown in this study included a decrease in the use of artificial tears, reduced conjunctival hyperemia, and improvement in tear secretion and tear film stability (Table 2) [45].

**Table 2:** Comparison of recent studies on fatty-acid supplementation for dry eye treatment

Study	Sample size	Dose	Study variables	Duration of study	Outcomes
Gatell-Tortajada [45]	1419 patients (74.3% women, mean age 58.9 years)	3 capsules/day of nutraceutical formulation (Brudysec^®^ 1.5 g)	Dry eye symptoms, conjunctival hyperemia, tear breakup time (TBUT), Schrimer I test, Oxford grading scheme	12-week prospective study	Symptoms improved significantly, artificial tear use decreased Schirmer test score, TBUT increased significantly, increase in patients grading 0–I in Oxford scale and decrease of those grading IV–V
Epitropoulos et al. [46]	105 subjects (ω-3 [n = 54]) (control [n = 51])	Randomized to receive 4 softgels containing 1680 mg of EPA/560 mg of DHA or control of 3136 mg of linoleic acid	Measure tear osmolarity, (MMP-9), (TBUT), (OSDI), fluorescein corneal staining, schirmer score, meibomian gland dysfunction (MGD) stage and ω-3 index	12 week prospective study measured at baseline, week 6, and week 12	Statistically significant reduction in tear osmolarity in the ω-3 group vs. control, increase in ω-3 index levels and TBUT ω-3 group significant reduction in MMP-9 positivity versus control group, OSDI scores decreased significantly in ω versus control group
Malhotra et al. [49]	60 patients with moderate MGD were allocated alternately to treatment and control groups	Both received warm compresses, lid massage, and artificial tear substitutes. Treatment group also received oral supplements of 1.2 g ω-3 FAs per day	To assess improving contrast sensitivity (CS) of patients with moderate meibomian gland dysfunction (MGD)	12 week prospective study. All parameters were recorded at baseline and at 12 weeks	Ocular surface disease index, tear break-up time, ocular surface staining, and meibum quality and expressibility improved significantly more so treatment group
Deinema et al. [50]	54 participants were randomized into 3 groups and received 1 of 3 interventions	Placebo (olive oil 1500 mg/day), krill oil (945 mg/day [EPA], + 510 mg/day [DHA], or fish oil (1000 mg/day EPA + 500 mg/day DHA) for 90 days, with monthly study visits	To assess the efficacy of 2 forms of oral long-chain ω-3 essential fatty acid supplements, phospholipid (krill oil) and triacylglyceride (fish oil), for treating DED	3 month prospective study, parameters were measured at baseline and at 90 days	Tear osmolarity reduced in krill oil and fish oil supplements, OSDI score was significantly reduced in krill oil group only, Relative improvements in tear breakup time and ocular bulbar redness, compared with placebo, for both forms of ω-3 EFAs

Epitropoulos et al. sought out to assess the effect of oral re-esterified ω-3 fatty acids on tear osmolarity, matrix metalloproteinase-9 (MMP-9), tear break-up time (TBUT), Ocular Surface Disease Index, fluorescein corneal staining, Schirmer score, MGD stage and ω-3 index in subjects with dry eyes and confirmed MGD (Table 2) [46]. The mean of 105 subjects who completed the study were 56.8 ± 17.0 years, of which 51% were randomized to the ω-3 group and 49% to the control group with 71.4% of the group being females [46]. Tear osmolarity plays a key role in determining the severity of DED; as a result, there was a significant reduction in osmolarity within this study at 12 weeks (P = 0.004) [46]. This study showed significant improvement in the TBUT scores and a significant reduction in subjects testing positive for MMP-9 bioenzyme in the tear film, which are both correlated with an improvement in DED [46]. An improvement in the TBUT scores indicates that the dietary supplementation with the ω-3 fatty acids improves the inherent stability of the tear film [46].

Sambursky and co-authors conducted a retrospective single center medical chart review of 100 patients that determined the effectiveness of using MMP-9, which is induced by key cytokines in the early stages of the inflammatory cascade, as an ideal biomarker because its elevation confirms the presence of clinically significant ocular surface inflammation [31]. MMP-9 is characterized as a proteolytic enzyme produced by stressed epithelial cells on the ocular surface in DED, which is elevated throughout the progression of DED [47, 48].

Malhotra et al. conducted a study that involved 60 patients with moderate MGD that were divided into a treatment group, composed of seventeen females and thirteen males aged 53.3 ± 6.9 years that received an oral supplementation of a triglyceride formulation of ω-3 fatty acids (FAs) and a control group, composed of eleven females and nineteen males aged 53.6 ± 8.7 years [49]. Artificial tear substitutes and eyelid hygiene was given to each group, which consisted of warm compressers and lid massage once daily for a period of 12 weeks (Table 2) [49]. Multiple improvements were noted including photopic and scotopic contrast sensitivity, tear film stability represented by the prolongation of the TBUT from baseline, an increase in tear secretion noted by the increased value of Schirmer score, and an improvement in ocular surface staining over the duration of the study from baseline to 12 weeks [49]. Deinema and co-authors revealed through a randomized, double-masked, placebo-controlled clinical trial with 54 participants that a moderate daily dose of krill oil (945 mg/day EPA, +510 mg/day DHA) and fish oil (1000 mg/day EPA + 500 mg/day DHA) for 3 months, resulted in improved symptoms in individuals with DED, including reduced tear osmolarity and increased tear stability (Table 2) [50].

### In vitro studies

Recent research has also indicated promising results through experiments performed in vitro through the use of human corneal epithelial [51] cells.

Erdinest and co-authors determined the anti-inflammatory effects of systemic PUFAs on HCE cells in vitro [52]. Studies revealed that topical anti-inflammatory therapies inhibit the various inflammatory mediators and reduce the signs and symptoms of DED [52]. Topical corticosteroids show efficacy in treating dry eye associated inflammation, and have a rapid onset of action [20]. In this study, cells were treated with inflammation inducers such as lipopolysaccharide (LPS) and LPS binding protein or with polyriboinosinic: polyribocytidylic acids at a dose of 25 µg/mL [52]. ALA dramatically reduced the poly I:C and LPS complex stimulated production of the pro-inflammatory cytokines TNF-α, IL-6, interleukin-1β (IL-1β), and the chemokine interleukin-8 (IL-8) in cultured HCE cells, specifically a dose-dependent reduction was demonstrated by ALA for all of these anti-inflammatory mediators [52]. ALA showed inhibitory effects on the protein secretion of the inflammatory mediators [52], and it also demonstrated a decrease in nuclear factor of kappa light polypeptide gene enhancer in B-cells inhibitor, alpha (Iκβα) mRNA expression, suggesting the anti-inflammatory effects of ALA involve regulatory effects of the NF-κβ pathway [52].

In the PUFAs family, there have been four new groups of pro-resolution mediators that have been identified which include lipoxins, protectins, maresins and resolvins (RVs) [18]. RVs are endogenous, potent, local acting molecules classified as non-classical Eicosanoids [18, 53]. RVs can be formed through metabolizing the compounds EPA and DHA [18, 54]. The E-series RVs are formed when EPA is metabolized first by cyclooxygenase-2 or the cytochrome-P450 pathway in vascular endothelial cells, and then by neutrophil 5-lipooxygenase [54]. Since inflammation plays a crucial role in the pathogenesis of DED, RVs could have significant therapeutic implications. Erdinest et al. looked at the efficacy of RV-D1 treatment in HCE cells in vitro after stimulation with poly I:C [55]. Results indicated a highly potent anti-inflammatory effect of RV-D1 on HCE cells in vitro [55]. RV-D1 significantly reduced the Poly I:C inflammatory reaction and dramatically reduced the production of the pro-inflammatory cytokines TNF-α, IL-6, IL-1β and IL-8 in cultured HCE cells [55]. A significant dose-dependent reduction was demonstrated by RV-D1 for TNF-α and IL-1β [55]. RV-D1 demonstrated inhibitory effects on the protein production of the inflammatory mediators [55]. Furthermore, the HCE cells stimulated by Poly I:C and treated with RV-D1 demonstrated a decrease in Iκβα mRNA expression, suggesting that the anti-inflammatory effects of RV-D1 involve regulatory effects of the NF-κB pathway [55].

### Future directions and challenges for dry eye disease

Dry eye disease is a relatively common condition in which the individual does not produce enough quality tears to lubricate and nourish the eye. Despite recent advances, advances in ocular surface lipid research for DED have been slow going due to numerous unanswered questions regarding the role of lipids. Once the lipidomic profiles of this disease has been characterized, it will provide vital answers to how lipid-based therapies are incorporated into overall anti-inflammatory strategies for the ocular surface. Furthermore, there are still limited options for lipid-based anti-inflammatory therapy. In the future, advances in identifying and quantifying lipids throughout this disease will provide a better understanding of ocular surface inflammation and allow clinicians to provide better treatments for DED.

## Diabetes mellitus

Diabetes mellitus (DM) is a common metabolic disease characterized as a hyperglycemic condition [56, 57]. In the United States, it is an epidemic disease where approximately 6.2 million people are underdiagnosed [56]. DM is separated into two main categories: type 1DM (T1DM) and type 2DM (T2DM). T1DM, or insulin dependent diabetes, is due to the autoimmune destruction of the β-cells in the pancreas [58, 59]. A prolonged excessive elevated blood glucose level that eventually leads to insulin resistance leads to T2DM, or non-insulin dependent diabetes [58, 59]. T2DM is the most prevalent in the United States, and it has seen significant rises in prevalence over the past thirty years.

The cornea suffers from a substantial amount of changes and injuries in DM due to reduced corneal sensitivity, increased corneal thickness, susceptibility to corneal trauma, persistent epithelial defects, corneal epithelial damage, recurrent corneal erosions, and alteration in tear quality and quantity [57, 60]. A severe complication of diabetes and leading cause of preventable blindness is diabetic peripheral neuropathy (DPN). DPN is a well-known microvascular complication of T2DM which leads to further infections and increases the risk of mortality; it occurs in a considerable amount of diabetic patients [61, 62]. As DM continues to become more prevalent across the world, the number of people at risk for developing DPN continues to increase. DM has a substantial effect on the ocular tissues even when it is well managed, which contributes to the disease severity when concerning the cornea. The major pathogenic factor of DM is hyperglycemia, which is a significant contributor to the accumulation of AGEs [13, 63]. Diabetic retinopathy is the most common ocular complication of diabetes, but there are many others including corneal dysfunction, cataracts, glaucoma, neuropathy, ischemic optic neuropathy, and diabetic macular edema [64, 65].

Many attempts with approved treatments have failed in clinical trials to slow progression or inhibit all of the complications associated with corneal DM. Recent studies have suggested the possibility of using lipid-based therapies to provide benefit to patients suffering from DPN, and research plays a key role in discovering novel treatments for these individuals. Recent advances in the use of lipids as novel therapeutics are discussed.

### Novel lipid-based therapeutics to treat diabetic neuropathy

Recent studies have determined one beneficial treatment for DPN is dietary enrichment with n-3 fatty acids. Coppey and co-authors used a rat model for T1DM with a dietary enrichment of menhaden oil, a natural source of n-3 fatty acids, that prevented and reversed multiple pathological endpoints associated with DPN [66]. Former studies have demonstrated that DM can cause the production and accumulation of free oxygen radicals, key factors contributing to the early development of DPN [67].

Corneal nerves in the subepithelial layer and epithelium are significantly decreased in diabetic rats indicating an excessive amount of damage to the nerve fibers [66]. However, upon treatment with a diet enriched with menhaden oil, a prevention and/or reversal in the loss of corneal nerves was observed in these diabetic rats indicating potential therapeutic benefits for this molecule [66]. Menhaden oil is used as a source of ω-3 (n-3) polyunsaturated fatty acids, enriched in EPA and DHA as a dietary supplement for human consumption [68]. Upon onset of hyperglycemia and the development of diabetic complications related to neuropathy, Shevalye et al. has shown that treatment of a mouse model suffering from T2DM with a dietary supplement of menhaden oil or daily injections of RV-D1 reverses neuropathic deficits [68]. This includes the slowing of nerve conduction velocity and decreased progression of loss of sensitivity and/or density of nerves in the skin, cornea, and retina [68]. RvE1 is also a potential anti-inflammatory therapy for patients with corneal inflammation [69].

Another potential treatment to patients suffering from DPN is the use of anti-inflammatory compounds such as salsalate [70]. Salsalate, a prodrug of salicylate that allows for better oral absorption, establishes its primary mechanism of action as a nonsteroidal anti-inflammatory which functions to inhibit the synthesis of prostaglandins through the inactivation of cyclooxygenase enzymes [71]. Research has indicated the usefulness of enalapril, an angiotensin converting enzyme inhibitor and α-lipoic acid, an antioxidant, in their ability to individually partially improve DPN [72]. More recent studies have indicated that the combination of enalapril, α-lipoic acid and menhaden oil treatment was able to fully reverse the neuropathic endpoints except for motor nerve conduction velocity [72].

### Lipid-mediated corneal neuroprotection

Docosahexaenoic acid is necessary for a multitude of functions in the body including memory formation, neuroprotection, synaptic function, as well as brain and retina development; it is involved in several processes, including photoreceptor biogenesis and function, excitable membranes functions, and photoreceptor biogenesis and function [73–80]. The importance of these (n-3) fatty acids in vision has been established through recent studies. One of the more characterized members of this family include neuroprotectin D1 (NPD1), which exhibits many beneficial effects throughout other parts of the body including reactive oxygen species (ROS) production and cyclooxygenase activities in human neutrophils [81–83]. Due to the anti-inflammatory effects it has shown in various parts of the body, it has been speculated for the potential use as a neuroprotectin within the cornea.

Docosahexaenoic acid is the precursor to the lipid mediator NPD1, which has shown to have potent anti-inflammatory actions [84, 85]. However, specialized pro-resolving mediators such as DHA and NPD1 have shown the ability to resolve inflammation without the mechanism of immune suppression, indicating the use of the term immunoresolvents [86, 87]. Recent studies have shown that corneas treated with DHA along with pigment epithelial-derived factor exhibit an increase in NPD1 synthesis, indicating the potential use of NPD1 as an effective treatment for neurotrophic corneas [18, 88]. An early inflammatory response is key for corneal wound healing, but it must be resolved to see proper tissue homeostasis [89]; as a result, Cortina et al. has indicated the effect of NPD1 on corneal nerves may be due to an injury to the cornea, leading to modulation of the inflammatory response, which leads to corneal epithelial cell survival [88]. These neuroprotectins offer interesting options for novel therapeutics in the treatment of DPN and other diseases involving corneal nerve damage.

### Future directions and challenges for diabetes mellitus

Corneal defects have been reported in both T1DM and T2DM patients, but the literature is currently lacking exact pathophysiological differences. DM involves increased corneal thickness [90, 91], reduced corneal sensitivity, corneal epithelial lesions [92, 93], delayed wound healing capacity and repair mechanisms [94, 95], and weakening of the epithelial barrier [96] leading to corneal infections and stromal fibrosis [97, 98].

Recent studies have shown that there are several metabolites that are differentially regulated in both T1DM and T2DM constructs when compared to human corneal fibroblasts (HCFs) as well as significant differences in mitochondrial structure in both T1DM and T2DMs, as compared to HCFs, when allowed to secrete and assembly their own extracellular matrix [60]. Future studies are needed to investigate the molecular mechanisms of human diabetic keratopathy in order to better understand corneal stromal defects due to DM and develop novel therapeutics to treat diabetic keratopathy defects.

## Fuchs’ endothelial corneal dystrophy

Fuchs’ endothelial corneal dystrophy (FECD) is a relatively frequent degenerative corneal condition confined to the corneal endothelium, characterized by subsequent changes in cell morphology, as well as deposits of collagen in the Descemet membrane, apoptosis, [99–101] and endothelial cell loss that is inherited in an autosomal dominant pattern with 100% penetrance with variable expressivity [102, 103]. FECD is estimated to affect approximately 4% of Americans older than 40 years old, but the exact incidence is unknown due to the fact that symptomatic FECD is preceded by an asymptomatic phase [104]. FECD is associated with a loss of Na^+^/K^+^-ATPase pump sites within the endothelium that eventually leads to corneal swelling [105, 106]. FECD is associated with blurred vision, which is caused by this corneal swelling from defects in the inner corneal layer, the corneal endothelium [104, 107].

The current definitive therapy is lacking, but it includes endothelial keratoplasty; furthermore, there are currently no available nonsurgical treatments to delay or prevent the progression of the disease [100, 108]. The disease typically presents with its clinical characteristics at some point between the 5th and 6th decades of life and can vary from macroscopic guttae producing light scattering to a decrease in visual quality, tochronic, and full thickness corneal edema [107]. Currently, the disease is considered to be a multifactorial condition, in which genetic [109, 110] and environmental factors, such as UV light-induced oxidative stress [100, 108, 111], contribute to its onset. Keratoplasty, specifically penetrating keratoplasty, has been the surgical approach of choice for many years, but currently endothelial keratoplasty such as Descemet membrane endothelial keratoplasty and Descemet-stripping automated endothelial keratoplasty are mainly performed for patients with FECD [112–114]. Currently, the primary cause of this endothelial dysfunction is unknown, but recent studies have attempted to classify changes associated with this disease.

### Lipidomic profiling in FECD

The potential to identify differences in lipids associated with diseases such as FECD has been greatly improved by recent technical advances in lipidomic research such as ultra-performance liquid chromatography mass spectrometry [115]. Aqueous humor (AH) composition has been suspected to play a role in the pathophysiology of FECD [116], but the precise role remains unclear. A recent study revealed key mediators in the lipidomic profile for individuals suffering from FECD indicating the lipid composition of the AH in FECD patients differs from that of healthy subjects. The concentration of most diacylglycerophosphocholines, 1-ether, 2-acylglycerophosphocholines, eight sphingomyelins, and up to two long-chain highly unsaturated cholesteryl esters increased in the AH of FECD eyes as compared to the healthy controls [117]. These differences may be due to oxidative stress-related changes in the lipid metabolism of the corneal endothelial cells in FECD, but this is one of the first studies published concerning lipidomic changes in FECD and was performed with an exploratory scope to screen for more specific works in the future [117]. The lipidomic profile of ten AH controls with an average age of 50.8 years and eight AH FECD patients with an average age of 56.8 years were analyzed in the study, indicating a need for expansion to verify results [117]. Richardson et al. looked at the varying differences of protein concentration between FECD individuals and control, indicating potential markers for the disease [118], but Cabrerizo et al. was the first study to look at the lipid profile of FECD individuals. Future studies could indicate a set of markers for the disease and enable detection before there is a deficit in vision.

### Nonsurgical treatments for FECD

Due to the lack of nonsurgical treatments to delay or prevent the onset of FECD, Kim et al. has sought out to screen drugs with corneal endothelial cell survival effects against two of the key physiologic markers of FECD, including oxidative stress and unfolded protein response (UPR) [119]. Multiple studies have described an association of oxidative stress-triggered UPR through various models including an endoplasmic reticulum stress-mediated apoptosis induced by cigarette smoke via a ROS dependent mechanism, an oxidative stress-triggered UPR in cell death evoked by Parkison mimetics, and the induction of UPR in a ROS through tumor necrosis factor-alpha [120–123]. Results from this study demonstrated a dose response effect of the non-steroidal anti-inflammatory drugs (NSAIDs), mefenamic acid and nimesulide, against oxidative stress and UPR in a bovine corneal endothelial cell (CEC) culture model [119]. Mefenamic acid was shown to significantly decrease oxidative stress in immortalized human corneal endothelial cells (iHCECs) exposed to H_2_O_2_ [119]. Nimesulide is a cyclooxygenase-2 inhibitor and acts as an antioxidant to correct lipid peroxidation products such as malondialdehyde [124] in addition to reversing UPR through the NF-κβ and pp38 kinase pathways [125]. Results indicate potential speculation for the use of NSAIDs such as mefenamic acid and nimesulide for the use as a survival factor for corneal endothelial cells undergoing oxidative stress and UPR [119].

Recent studies have also looked at the potential use of corneal regenerative medicine using cultured endothelial cells, which includes mesenchymal stem cell derived conditioned media as well as Rho-associated protein kinase inhibitors [126–128]. The development of a successful and reliable cell cultivation protocol for clinical application has drastically slowed the establishment of tissue engineering-based therapy for corneal endothelial dysfunction [129]. However, many studies have characterized the successful transplantation of a cultured corneal endothelial sheet into an animal model [126, 130–133]. Studies have demonstrated that Rho–ROCK signaling negatively regulates the integrin-mediated adhesion of monocytes, and that the inhibition of ROCK by a selective ROCK inhibitor upregulates adhesion, showing potential for enhancing the adhesion of CEC by inhibiting Rho/ROCK signaling [128, 134, 135]. Rho–ROCK signaling is involved in a multitude of cellular processes including migration, morphogenesis, cell adhesion, and cell-cycle progression through mediating cytoskeletal dynamics [136, 137].

These studies promote the potential for using ROCK inhibitor Y-27632 to enable the establishment of a cultivated-CEC-based therapy by inhibiting apoptosis, increasing the number of proliferating cells, and promoting the adhesion through a rabbit and primate corneal endothelial dysfunction model [127, 128]. This novel strategy may ultimately provide clinicians with a potential treatment for FECD, through the use of a ROCK inhibitor using regenerative medicine [127].

### Future directions and challenges for Fuchs’ endothelial corneal dystrophy

Investigation into FECD has revealed a better understanding of this disease at the tissue, cellular, and molecular level. Despite substantial research, the literature still lacks consistent markers for this multifactorial disease.

Cabrerizo and co-authors have revealed the potential use of various lipids as biomarkers for the severity of disease in FECD [117], indicating promise for a better understanding of the pathophysiology of this disease. Corneal endothelial dysfunction is a substantial problem in FECD, and it is accompanied by visual disturbance which provides a major indication for corneal transplantation surgery [138]. Descemet’s stripping endothelial keratoplasty is a highly effective surgical technique designed to replace corneal endothelium and overcome pathological dysfunctions of corneal endothelial tissue [139–141]. Corneal transplantation is still widely used for corneal endothelial dysfunction, but the transplantation of cultivated corneal endothelium is a new potential therapeutic strategy [127].

Studies involving lipids reveal the potential use of NSAIDs [119] for improving symptoms associated with FECD. The need for nonsurgical treatments for FECD continues to be a problem despite recent advances, indicating the need for future studies for novel lipid-based therapeutics.

## Concluding remarks

The health of the ocular surface is crucial for the patient’s quality of life, and it may be compromised through diseases or dystrophies. There are still limited options for lipid-based therapies, but there have been numerous advances over the past few years in novel lipid-based therapeutics for the treatments of these diseases. Lipids also show increasing promise as biomarkers for diseases such as FECD indicating increasing clinical relevance. Immunomodulation through lipids such as essential fatty acids has shown promising results as therapeutic molecules.

Despite significant progress, further studies concerning the role of lipids and the exact mechanism of action must be classified. However, lipid-based therapies provide encouraging results for the treatment of these diseases and dystrophies.

## Abbreviations

DED: dry eye disease
AGE: advanced glycation end products
EPA: eicosapentaenoic acid
DHA: docosahexaenoic acid
ALA: alpha linolenic acid
PUFA: polyunsaturated fatty acid
IHMGECs: immortalized human meibomian gland epithelial cells
MMP-9: metalloproteinase-9
TBUT: tear break-up time
MGD: meibomian gland dysfunction
LPS: lipopolysaccharide
DM: diabetes mellitus
T1DM: type 1DM
T2DM: type 2DM
DPN: diabetic peripheral neuropathy
NPD1: neuroprotectin D1
FECD: Fuchs’ endothelial corneal dystrophy
AH: aqueous humor
CEC: corneal endothelial cell
UPR: unfolded protein response
NF-κβ: nuclear factor kappa B
LPS: lipopolysaccharide
HCFs: human corneal fibroblasts
TNF-α: tumor-necrosis factor-α
TGF-β: transforming-growth factor-β
IL-1β: interleukin-1β
IL-6: interleukin-6
IL-8: interleukin-8
IL-1α: interleukin-1α
FAs: fatty acids
Iκβα: nuclear factor of kappa light polypeptide gene enhancer in B-cells inhibitor alpha
RVs: resolvins
ROS: reactive oxygen species
IL-10: interleukin-10
IL-8: interleukin-8
IL-1β: interleukin-1β
NSAIDs: non-steroidal anti-inflammatory drugs
